# Clinical Study on the Efficacy and Safety of Arginine Administered Orally in Association with Other Active Ingredients for the Prevention and Treatment of Sarcopenia in Patients with COVID-19-Related Pneumonia, Hospitalized in a Sub-Intensive Care Unit

**DOI:** 10.3390/healthcare10010162

**Published:** 2022-01-14

**Authors:** Carolina Bologna, Eduardo Pone

**Affiliations:** 1 Internal Medicine Ospedale del Mare di Napoli, 80100 Naples, Italy; 2 Medicina d’Urgenza Ospedale del Mare di Napoli, 80100 Naples, Italy; eddi22@alice.it

**Keywords:** COVID-19, SARS-CoV-2, arginine

## Abstract

In order to evaluate the efficacy of oral supplementation with 3 g of arginine per day associated with creatine, L-carnitine, aspartic acid, magnesium, selenium and vitamins C and E (Argivit© Aesculapius Farmaceutici) in the prevention and treatment of sarcopenia in patients with COVID-19-related pneumonia, we conducted a parallel randomized study comparing it with standard therapy alone. Forty patients on standard therapy plus supplementation were compared with a control group of 40 patients, all hospitalized at the sub-intensive care unit of the Del Mare Hospital in Naples, with a clinical diagnosis of SARS-CoV-2 infection and COVID-19 pneumonia. Muscle strength was assessed with the handgrip test and muscle ultrasound. Arginine-supplemented patients had an average grip strength of 23.5 at the end of hospitalization compared with 22.5 in the untreated group with less reduction, showing statistical significance (*p* < 0.001). In the same way, the thickness of the vastus lateralis quadriceps femoris muscle measured at the end of hospitalization showed less reduction on ultrasound, with a higher average value in the group receiving treatment than in the group of patients without supplementation (*p* < 0.001). Upon discharge there was a 58.40% reduction in ventilation days in patients with arginine supplementation compared with the control group.

## 1. Introduction

L-Arginine is an amino acid classified as essential because it plays a key role at certain times of life (such as adolescence, puberty, extensive trauma, burns). Supplementation with L-arginine stimulates nitric oxide production, improves collagen synthesis in tenocytes, improves tendon function and accelerates healing of damaged tendons. In our COVID Operative Unit, the use of arginine, in association with other important active principles from the metabolic, energetic and immunological points of view, among others, such as creatine, L-carnitine, aspartic acid, magnesium, selenium and vitamins C and E (Argivit© Aesculapius Pharmaceutics, Brescia, Italy), has made it possible to count on several different and synergic actions, and in particular the anabolic and pro-energetic effect on striated muscles, useful in improving respiratory dynamics and supporting the endothelium in the production of nitric oxide, starting with its only precursor, arginine. Nitric oxide released by endothelial cells, neutrophil granulocytes and macrophages causes vasodilation, reduces platelet aggregation and inhibits several aspects of mast cell-induced inflammation and leukocyte recruitment. NO production in skeletal muscle also contributes to improved metabolic control by altering blood flow, glucose uptake, oxidative phosphorylation, contractility and excitation–contraction coupling. NO saves metabolic reserves by promoting glucose uptake and inhibiting glycolysis, mitochondrial respiration and phosphocreatine consumption. Creatine supplementation, in addition to enhancing thermoregulation, improves post-concussion rehabilitation and/or neuroprotection of the spinal cord [1,2].

In the context of a complex and multifactorial disease, such as respiratory failure in hospitalized and defaulter patients, preventing sarcopenia and counteracting the general catabolic state is fundamental as it affects quality of life, complications and prognosis, also because at the same time it is essential to act on respiratory dynamics. In the prevention of sarcopenia in bedridden patients, linked to long periods of inactivity and immobilization, arginine is also effective in improving respiratory dynamics. L-Arginine supplementation should be at least 3 g per day.

Recent studies carried out in China and Italy [3,4] show with certainty that problems related to fatigue and tiredness persist in patients who recovered from COVID-19 even after months. This becomes the basis for the use of arginine in association with other active ingredients, important from the point of view of energy balance, in these abstinent patients who also show persistent respiratory difficulties. This is a significant proportion of patients, 5–6 patients out of 10. The study conducted in China in patients who recovered from COVID-19 reported anxiety or depression in 23% of patients; in the Italian study anxiety and depression were 11.3% and 4.5%, respectively [3,4]. The effect expected from the administration of arginine in association with other active ingredients on functional performance could improve these conditions and positively influence the symptoms in the psychological field as well. Based on the literature, there are no risks with a dose of 3 g per day [5,6].

A study by Professor Santulli’s group in 2020 showed advantages in combining L-arginine with other substances, such as vitamin C. Vitamin C is known to significantly reduce the permeability of the endothelial barrier, an aspect which has important implications in infections, which cause a systemic increase in oxidative stress. Therefore, the antioxidant roles of vitamin C and its protective effects on endothelial permeability could be beneficial, especially during infectious processes. Vitamin C has also been shown to act synergistically with other substances: for example, L-arginine supplementation has been shown to play a favorable role in regulating immune responses. In a similar way, both vitamin C and L-arginine improve endothelial function and reduce vascular permeability during infections. Consequently, it is reasonable to hypothesize that their association may be synergistic in the treatment of infectious diseases, such as in the case of COVID-19, which causes severe endotheliopathy [7].

Fiorentino and colleagues recently published a parallel-group, double-blind, randomized, placebo-controlled trial conducted on patients hospitalized for severe COVID-19. Patients received 1.66 g L-arginine twice a day or placebo, administered orally. The primary efficacy endpoint was a reduction in respiratory support assessed 10 and 20 days after randomization. Secondary outcomes were the length of in-hospital stay, the time to normalization of lymphocyte number, and the time to obtain a negative RT-PCR test for SARS-CoV-2 on nasopharyngeal swab. Results of this initial interim analysis on the first 101 patients showed that, at 10-day evaluation, 71.1% of patients in the L-arginine arm and 44.4% in the placebo arm (*p* < 0.01) had the respiratory support reduced; however, a significant difference was not detected 20 days after randomization. Strikingly, patients treated with L-arginine exhibited a significantly reduced in-hospital stay vs. placebo, with a median (interquartile range, 25th–75th percentile) of 46 days (45–46) in the placebo group vs. 25 days (21–26) in the L-arginine group (*p* < 0.0001). The other secondary outcomes were not significantly different between groups [8]. From a pathophysiological point of view, it is well known that in COVID-19 infection, consecutive inflammatory cell recruitment and endothelial dysfunction may explain the impaired microcirculation observed across vascular beds, triggering vasoconstriction, ischemia and a pro-coagulant state [9]. Therefore, endotheliitis has been suggested as the major cause of systemic impaired microcirculatory function observed in different vascular beds in COVID-19 patients. It was shown that arginine protects cardiac, hepatic, intestinal and pulmonary microcirculation from ischemia and reperfusion damage [10], and both endothelial dysfunction and T cell impairment are consequences of low L-arginine bioavailability and can contribute to complications of COVID-19. Consequently, the role of L-arginine supplementation is biologically plausible, and the above-mentioned results may have important clinical implications for COVID-19 treatment [8].

In Santulli’s study [7] it was also reported that many experimental data indicate, in fact, that arginine improves immune function, whereas its deficiency alters the immune response and therefore may increase the risk of healthcare-associated infections. In a study in a porcine model with endotoxemia, L-arginine supplementation improved not only immune response but also protein turnover and resulted in elevated NO synthesis throughout the body, with no adverse effects [11]. It was also hypothesized that arginine supplementation could reverse the immunocompromised state.

The aim of our study was to evaluate the benefit and efficacy of arginine in the prevention and treatment of sarcopenia in COVID-19 patients compared to a group with standard treatment without arginine supplementation.

## 2. Materials and Methods

### 2.1. Patient Selection

We performed a retrospective analysis on patients admitted to COVID-19 sub-intensive care unit of Ospedale del Mare that performed standard care for COVID-19 and supplementation of arginine when required. With this focus, we selected 40 patients that took supplementation of arginine and 40 patients that did not require it.

Patients that required supplementation were patients with advanced chronic illness and/or that were selected for long-term hospitalization for COVID-19 and/or with low BMI/defedated patients.

In order to escape misunderstanding regarding defedated patients, we performed muscle mass evaluation with objective methods.

Traditionally, muscle mass is measured by computed tomography (CT) or dual-energy X-ray absorptiometry (DEXA). These devices are not always readily available in clinical practice, but above all they cannot be used at the patient’s bedside. In particular, this was not feasible in the COVID patient. Therefore, muscle mass assessment was carried out using the ultrasound scanner, which is available and applicable in all patients. The ultrasound of the vastus lateralis muscle had a scan point on the front of the thigh at a distance of one-third from the superior patella to the anterior superior iliac spine. It was not possible, within the limits of the study resources, to have multiple ultrasound technicians perform each assessment to examine the reliability of the technician assessors in this study. To minimize the risk of measurement errors between operators, the follow-up scans were completed by the same ultrasound technician who had performed the baseline assessment. Scans were measured in triplicate on the thigh, and the average value was calculated. The change in muscle thickness was assessed as a secondary outcome for acute muscular atrophy.

The dominant leg was chosen to calculate the relative values of muscle thickness and length according to the proposals of the SARCUS working group [12]. The first assessment was performed as soon as possible within 48 h of admission, and the final assessment was performed on the last day before discharge.

Muscle strength was assessed using handgrip test or HGS grip strength: it was measured bilaterally with three attempts per hand using a Jamar digital dynamometer according to the Southampton protocol [1]. Several works suggest that grip strength is a useful indicator for general health status, early mortality from all causes, cardiovascular mortality and disability [13].

### 2.2. Objectives

The primary objective of our study was to evaluate the benefit and efficacy of arginine in the prevention and treatment of sarcopenia in COVID-19 patients compared to a group with standard treatment without arginine supplementation. For this purpose, the following parameters were defined:

- Sarcopenia assessment (ultrasound and handgrip test);
- Duration of non-invasive ventilation;
- Length of stay in the semi-intensive care unit (in days);
- Duration of stay at hospital (in days);
- Body mass index reduction
- Rate of transfer to intensive care unit;
- Mortality rate.

As secondary endpoints, the following were assessed:

- Time elapsed from admission to the sub-intensive care unit until functional goals were achieved;
- Characteristics of admission, including duration of ventilation;
- Length of stay in the sub-intensive care unit;
- Duration of hospital stay in acute care unit.
  Safety endpoints included treatment-emergent adverse events, serious adverse events and premature discontinuation of the study supplement.

Inclusion criteria were as follows:

- Patients of both genders, aged over 18 years, hospitalized at the COVID sub-intensive care unit of the Del Mare Hospital in Naples;
- Clinical diagnosis of SARS-CoV-2 infection confirmed by PCR or other approved diagnostic methodology;
- COVID-19-induced pneumonia documented by chest X-ray or CT scan with evidence of pulmonary infiltrates requiring oxygen supplementation and non-invasive ventilation on admission.

Excluded patients were as follows:

- Those who needed invasive mechanical ventilation on admission;
- Those with severe hepatic and renal impairment; severe heart disease; severe dementia syndrome; or coma, advanced terminal conditions, imminent death or highly probable death within 24 h;
- Those with leg edemas, anasarca due to severe renal failure or cirrhosis of the liver or severe dehydration or systemic connective tissue disorders, myositis, calcification and ossification of muscle, systemic atrophies mainly affecting the central nervous system or demyelinating diseases of the central nervous system.
  Other exclusion criteria were use of chronic oral corticosteroids before admission, history of intolerance to L-arginine, pregnancy or lactation and refusal to provide written informed consent.

Time 0 (T0) was taken as the date of admission to the care unit, and T1 was taken as discharge or transfer to another care unit.

The study obtained ethics committee approval (29 June 2021 Prot.904/CE22-2021 ASL Na1).

### 2.3. Statistical Analysis

The methods of descriptive statistics were applied to calculate the position and dispersion indices. In particular, normal continuous variables were expressed in terms of mean and standard deviation (SD). Category variables were expressed in terms of frequencies.

The comparison of the main continuous variables between the two treatment groups was carried out by means of the t-test or the equivalent non-parametric Mann–Whitney test.

The results of the Wilcoxon test for paired data on the differences between the start and end of therapy were added for significance.

A *p*-value < 0.05 was considered statistically significant.

Statistical analyses were performed using R software version 3.6.1 for Windows 10.3 (IBM ILOG SPSSv.25, Rome, Italy).

## 3. Results

Between 1 May 2020 and 30 June 2021, 160 patients were screened, of which 80 were eligible and admitted to the care unit. Patients received the study treatments for an average period of 21 days. Eighty patients completed the study; three patients in the treated group vs. six patients in the untreated group required transfer to the intensive care unit. Three patients in the treated group vs. six patients in the untreated group died.

We compared the two groups, the one treated with arginine associated with creatine, L-carnitine, aspartic acid, magnesium, selenium and vitamins C and E (Argivit© Aesculapius Farmaceutici, Brescia, Italy) versus the untreated one, in terms of the maintenance of grip strength (handgrip test) from the moment of admission (T0) and to the day of discharge (T1).

Table 1 shows hematochemical data of renal function, liver function, hemochromocytometric test and inflammatory indices, which were homogeneous in both groups. Regarding kidney functions, we found in the control group a slight increase in urea blood levels compared to the treated group, but this dysfunction was not joined to a similar increase in creatinine blood values, which are a more specific and reliable mirror of kidney activities.

**Figure 1:**
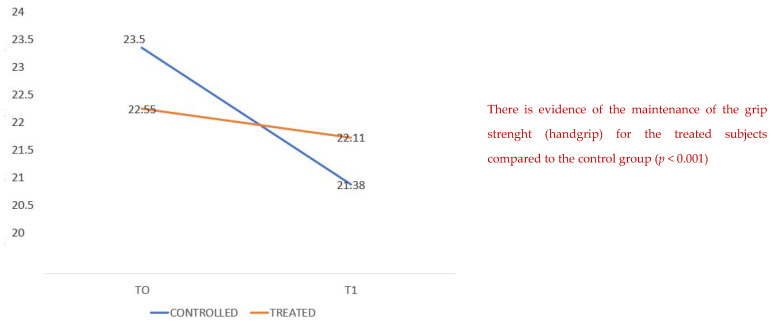
Handgrip test at T0 and T1. There is a better control of the grip strength (handgrip) for the treated subjects compared to the control group (*p* < 0.001).

The treated patients presented a better grip strength maintenance with a mean of 23.5 compared to 22.5 (Figure 1) for the untreated group with a statistical significance (*p* 0.001). The means of the blood urea values at baseline are higher in the treatment group, but from a clinical point of view the data are not relevant; moreover, the values of blood creatinine, a much more sensitive and reliable test, do not show clinically and statistically significant differences.

**Figure 2:**
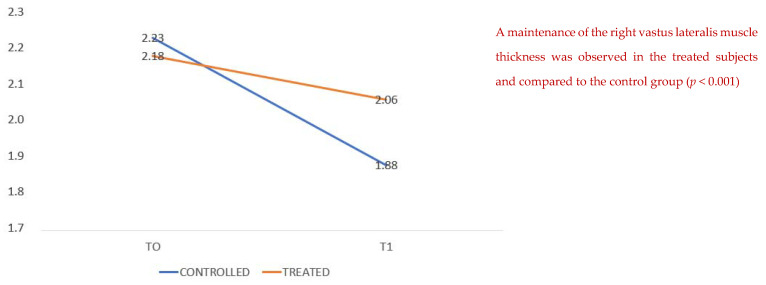
Strength assessment: right vastus lateralis muscle thickness. A maintenance of the right vastus lateralis muscle thickness was observed for the treated subjects compared to the control group (*p* < 0.001).

We compared the thickness of the vastus lateralis muscle at admission and at discharge, showing a higher average value in the treated group than in the untreated group (Figure 2); that is, patients with arginine supplementation associated with creatine, L-carnitine, aspartic acid, magnesium, selenium and vitamins C and E (Argivit© Aesculapius Farmaceutici) presented on average at the end of hospitalization (T1) a reduced loss of muscle mass as measured by ultrasound.

**Figure 3:**
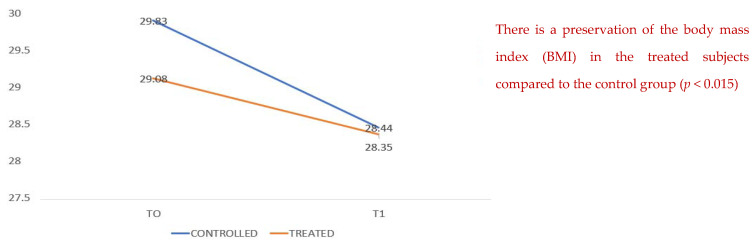
BMI T0 and T1. There is a conservation of the body mass index (BMI) for the treated subjects compared to the control group (*p* < 0.015).

Figure 3 compares data on body mass index, which show a decrease in both groups during hospitalization; however, it can be seen that in the treated group there is better conservation with less weight loss. There is a conservation of the body mass index (BMI) from T0 to T1 for the treated subjects compared to the control group (*p* < 0.015).

**Figure 4:**
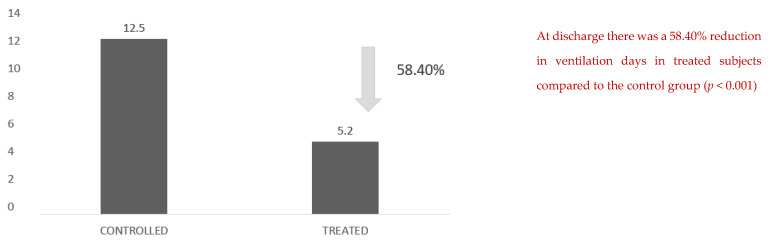
Days of ventilation at discharge. There was a 58.40% reduction in ventilation days in treated subjects compared to the control group (*p* < 0.001).

Another figure with an important clinical impact is the reduction in effective days of ventilation: there was a 58.40% reduction in days of ventilation in the treated subjects compared with the control group (Figure 4).

**Figure 5:**
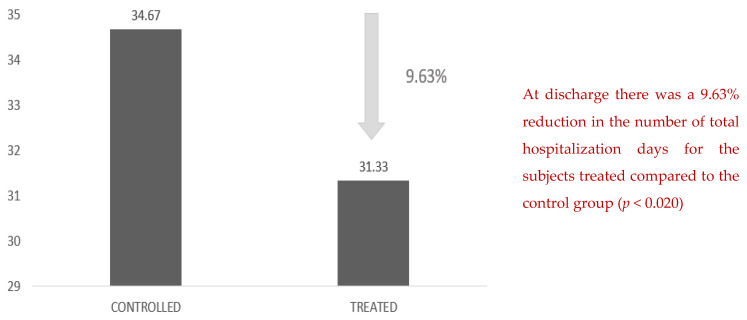
Number of total days of hospitalization. At discharge there was a 9.63% reduction in the number of total hospitalization days for the treated subjects compared to the control group (*p* < 0.020).

In the same way, there was a 9.63% reduction in the number of total hospital days for the patients treated compared with the control group (Figure 5).

The most clinically significant fact is the duration of hospitalization in the sub-intensive care unit: in the treated group, there was a reduction of 39.65% compared with the untreated group, with an average of 20.1 days versus 12.13 days. For length of stay in a sub-intensive care unit (days) in the treated group, there was a reduction of 39.65% compared with the untreated group, with an average of 20.1 days versus 12.13 days.

**Figure 6:**
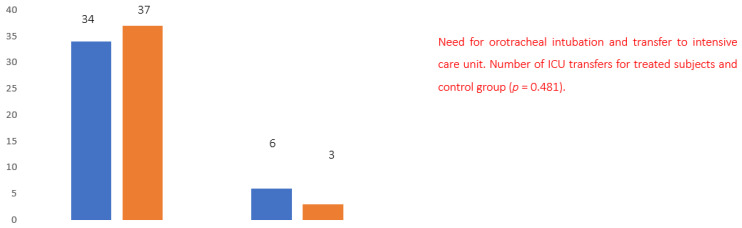
Need for orotracheal intubation and transfer to intensive care unit. Number of ICU transfers for treated subjects and control group (*p* = 0.481).

Evaluating the number of patients who showed a rapid worsening of the clinical picture requiring orotracheal intubation and transfer to the intensive care unit, six patients in the control group versus three patients in the treated group were reported to require intubation and transfer, that is, twice the number of cases (Figure 6).

## 4. Discussion

Even within the limits of the small number of patients observed, the data are very comprehensive: the improved muscular performance, objectively measured by grip strength and lateral rectus diameter using ultrasound, shows that supplementation with 3 g of arginine associated with creatine, L-carnitine, aspartic acid, magnesium, selenium and vitamins C and E (Argivit© Aesculapius Farmaceutici) not only results in a better prognosis for these patients, significantly reducing the length of stay in sub-intensive care unit and total hospitalization, but also shows an advantage in terms of mortality.

The anabolic and pro-energetic effect of arginine on striated muscles is useful in improving respiratory dynamics and supporting the endothelium in the production of nitric oxide. NO production in skeletal muscle also contributes to improved metabolic control by altering blood flow, glucose uptake, oxidative phosphorylation, contractility and excitation–contraction coupling. In the context of a complex and multifactorial disease, such as respiratory failure in hospitalized and defaulter patients, preventing sarcopenia and counteracting the general catabolic state is fundamental as it affects quality of life, complications and prognosis, also because at the same time it is essential to act on respiratory dynamics.

The main objective of our study was to evaluate the benefit and efficacy of arginine in the prevention and treatment of sarcopenia in COVID-19 patients compared to a standard treatment group without oral arginine supplementation. To this end, we demonstrated a minor reduction in muscle mass and muscle performance in the treated group. We demonstrated that treated patients had a shorter duration of non-invasive ventilation and consequently a shorter duration length of stay in the semi-intensive care unit (in days), as well as a shorter length of stay in total (in days); the treated group presented a minor reduction in body mass index and an ICU transfer rate for NIV failure with higher mortality in the untreated group.

Safety endpoints included treatment-emergent adverse events, serious adverse events and premature discontinuation of the study supplement. There were no adverse events or other noteworthy complications during the study.

## 5. Conclusions

We believe that nutritional support with this arginine-based supplement was essential to the improved muscular and respiratory performance of these patients, which was demonstrated by the reduction in the need for respiratory support. Further clinical investigations with larger sample sizes are undoubtedly needed, but this study suggests greater attention is needed in the assessment, prevention and treatment of sarcopenia and malnutrition in COVID patients undergoing non-invasive ventilation.

## Figures and Tables

**Table 1:** Clinical parameters at T0.

Clinical Parameters	Controls	Treated	*p* Value
BLOOD CREATININE	1.61	1.37	*p* = 0.065 (ns)
BLOOD UREA	87	64.45	*p* = 0.007
HEMOGLOBIN	11.02	10.98	*p* = 0.788 (ns)
GLYCEMIA	142.53	112.55	*p* = 0.019
SGOT	44.8	48.23	*p* = 0.942 (ns)
SGPT	47.38	47.88	*p* = 0.718 (ns)
Hs-CRP	15.65	19.45	*p* = 0.636
DURATION OF HIGH FLOW (DAYS)	7.71	7.14	*p* = 0.43 (ns)

ns = not significant.

## Data Availability

The data that support the findings of this study are available from the corresponding author, upon reasonable request.
